# Effects of Upper Limb Robot-Assisted Rehabilitation Compared with Conventional Therapy in Patients with Stroke: Preliminary Results on a Daily Task Assessed Using Motion Analysis

**DOI:** 10.3390/s23063089

**Published:** 2023-03-13

**Authors:** Marco Germanotta, Laura Cortellini, Sabina Insalaco, Irene Aprile

**Affiliations:** IRCCS Fondazione Don Carlo Gnocchi ONLUS, 50143 Florence, Italy

**Keywords:** rehabilitation, stroke, upper extremity, robotics, kinematics, movement analysis, stereophotogrammetry, recovery, motor impairment

## Abstract

Robotic rehabilitation of the upper limb has demonstrated promising results in terms of the improvement of arm function in post-stroke patients. The current literature suggests that robot-assisted therapy (RAT) is comparable to traditional approaches when clinical scales are used as outcome measures. Instead, the effects of RAT on the capacity to execute a daily life task with the affected upper limb are unknown, as measured using kinematic indices. Through kinematic analysis of a drinking task, we examined the improvement in upper limb performance between patients following a robotic or conventional 30-session rehabilitation intervention. In particular, we analyzed data from nineteen patients with subacute stroke (less than six months following stroke), nine of whom treated with a set of four robotic and sensor-based devices and ten with a traditional approach. According to our findings, the patients increased their movement efficiency and smoothness regardless of the rehabilitative approach. After the treatment (either robotic or conventional), no differences were found in terms of movement accuracy, planning, speed, or spatial posture. This research seems to demonstrate that the two investigated approaches have a comparable impact and may give insight into the design of rehabilitation therapy.

## 1. Introduction

According to the World Health Organization (WHO), cerebral stroke is the second leading cause of death, the third leading cause of disability globally, and the first leading cause of disability in the elderly. Concerning the upper limb, the prevalence of disability is approximately 50–80% in the acute phase [1,2] and up to 50% in the chronic phase [1,3]. Paresis, loss of dexterity and coordinated movements, abnormal muscle tone, and reduced sensation are the biggest contributors to the loss of upper limb function after stroke [4,5]. In addressing the concerns of such patients, motor function is the major evaluative metric and the most pressing issue [6]. Improving the motor function in patients is essential for enhancing their quality of life, lowering social and economic costs, and reducing the incidence of impairment. With this aim, after a stroke, rehabilitation treatment is routinely utilized [6].

Robot-assisted therapy (RAT) has been suggested as a promising method for upper limb rehabilitation, with the goals of increasing therapy volume and intensity, and standardizing the treatment [7]. In addition, robotic devices can quantify the patients’ dexterity thanks to the sensors and actuators they include [8,9]. Nowadays, upper limb RAT is recommended in several guidelines [10]. Numerous studies have been published on the topic of upper limb RAT for post-stroke patients, showing promising results for improving arm function, arm muscle strength, and the activities of daily living [11,12]. According to a very recent synthesis of the available systematic reviews [13], RAT had beneficial impacts on motor function and muscular strength, while there was no consensus on muscle tone effects, and just one systematic review reported a good impact of RAT on daily living activities. Therefore, RAT may be a beneficial choice for improving upper limb motor function and muscular strength. However, the low quality of the evidence may reduce confidence in the findings, requiring more studies on the topic. When compared to conventional approaches, recent randomized controlled trials (RCT) on large samples [14,15] failed to demonstrate the superiority of RAT over traditional approaches in the rehabilitation of motor performance in the upper limb.

Regarding the effect of RAT on the recovery of the ability to perform daily living activities, it is important to note that this is typically evaluated using validated clinical scales, which assess these abilities using ordinal scores that do not adequately account for how patients perform a particular task. In addition, clinical evaluations have limited sensitivity and reliability, as well as floor and ceiling effects. To this aim, the use of motion capture systems can help physicians and physiotherapists to understand and quantitatively evaluate the actual way the patient reacquires a particular skill, for example by using different strategies from those used by healthy subjects, or by optimizing the acquired strategies.

Several studies investigated upper limb kinematics during different tasks, mainly reaching tasks [16,17,18,19], while a few investigated more ecological tasks, linked to the activities of daily living. Among them, the drinking task was often selected because it was a task already learned, easy to standardize, and did not require specific devices [17]. A great contribution to the topic was made by Murphy et al. [20,21,22,23], investigating this task in post-stroke patients. Specifically, they analyzed the discriminant ability and sensitivity of the kinematic variables measured during a drinking task in patients with stroke [20], as well as their responsiveness in the acute [21] and chronic phases [22,23]. Using a similar task, Aprile et al. [24] focused on the strategies adopted in subacute stroke patients, in terms of arm elongation, trunk forward inclination, and trunk axial rotation, when compared to healthy individuals.

However, to the best of our knowledge, very few studies have been carried out in post-stroke patients using instrumented evaluation to determine if RAT is more effective than conventional therapy in restoring the motor skills of the upper limb while executing a task relevant to the activities of daily living. Therefore, this preliminary study aims to compare the improvement in motor skills in patients with stroke undergoing either a robotic or conventional treatment, through kinematic analysis of a drinking task.

## 2. Materials and Methods

### 2.1. Participants

In this study, we analyzed data from 19 patients with stroke, enrolled in a large, multicenter randomized controlled trial (clinicaltrial.gov identified: NCT02879279; ethics committee approval: FDG_6.4.2016). The trial was aimed at comparing upper limb robotic rehabilitation (using a set of four robotic and sensor-based devices) with conventional treatment. For the trial, we recruited consecutive subjects with the following inclusion criteria: a first-ever ischemic or hemorrhagic stroke that occurred between 2 weeks and 6 months before enrollment; age ranging from 40 to 85 years; upper extremity Fugl-Meyer Assessment motor function score ranging from 0 to 58. Exclusion criteria were behavioral and cognitive disorders and/or reduced compliance, fixed contraction in the affected limb, or significant visual acuity impairments.

For this study, among patients enrolled for the RCT in one center only, we analyzed those able to perform the required task, i.e., able to perform a reaching and grasping movement. No constraints on the strategies and the compensatory movement were imposed.

### 2.2. Rehabilitation Intervention

According to the randomization list for the main trial, patients were assigned to the Robotic Group (RG) or the Conventional Group (CG). More details on the treatments are reported elsewhere [15]. In the RG, patients underwent an upper limb robotic treatment with a set of four devices (Motore, Humanware, Stuart, FL, USA; and Amadeo, Diego, and Pablo, from Tyromotion, Graz, Austria) [25,26]. Motore is a robotic device that allows passive, active, and active-assistive planar movements in the shoulder and elbow joints; Amadeo is a robotic device that allows passive, active, and active-assistive finger flexion and extension movements; Diego is an electromechanical system that allows three-dimensional, unimanual, and bimanual movements of the shoulder joint with arm weight support; Pablo is a sensor-based system that allows unsupported three-dimensional unimanual and bimanual movements of the shoulder, elbow, and wrist joint. During therapy, patients completed both physical and cognitive activities, with visual and audio feedback given with the devices. In addition, vibratory therapy was administered using the Amadeo system to enhance the hand’s proprioception prior to finger training. Throughout each session, the physical therapist used a single system for each subject to reduce the amount of time necessary to transport the individuals between systems. Flowcharts with general guidelines were included in the chosen protocol in order to maintain treatment uniformity. Nonetheless, the physical therapist chose and tailored the workouts, in terms of workspace and difficulty, to the subject’s residual capacity. During the therapy, one therapist supervised a group of three patients.

In the CG, patients received conventional therapy with a ratio of one therapist to one subject. The treatment was based on the guidelines provided in the literature. Functional improvement was the objective of the therapeutic task, which included task-oriented exercises, sensorimotor restructuring, and spasticity inhibition. Subjects underwent passive, active, and active-assisted training on the three upper limb joints in 3D space to enhance joint function, avoid contractures, suppress spasticity, and enhance motor function. Functional improvement, sensorimotor rearrangement, and spasticity inhibition were the focal points of the therapeutic task. In addition, they performed task-oriented exercises, such as reaching and grasping movements and activities of daily living to increase the subject’s participation in order to promote neuroplasticity and enhance upper limb motor recovery. Each therapist was free to adapt every rehabilitation session to the subject, according to their functional assessment and needs.

The therapy (robotic or conventional) was administered daily for 45 min, 5 times each week, for 30 sessions. All patients also received the same dose of individual traditional physiotherapy for lower limbs.

### 2.3. Clinical Evaluation

In the main trial, several clinical outcome measures were used, selected according to the International Classification of Functioning, Disability, and Health. For the present study, we reported: the Fugl-Meyer Assessment for Upper Extremity (FMA-UE) (motor function) [27]; the upper-extremity subscale of the Motricity Index (strength) [28]; and the Modified Barthel Index (functional independence) [29].

### 2.4. Instrumental Evaluation

For this study, we investigated the ability of patients to perform a drinking task. The instrumental evaluation was carried out at the Movement Analysis Laboratory of the Fondazione Don Carlo Gnocchi in Rome, using an 8-camera stereophotogrammetric system working in the infrared range and equipped with CCD sensors and appropriate optical filters (Smart D500, BTS Bioengineering, Milan, Italy). The system recorded the three-dimensional coordinates of reflecting markers placed on anatomical landmarks on the human body. The system’s calibrated volume was about (3 × 3 × 2) m^3^, within which the 3D coordinates of retroreflective markers could be reconstructed with an accuracy of less than 1 mm in all directions. Before each acquisition session, the cameras were calibrated according to the normal technique following the standard procedure described by the producer of the motion capture system. In this study, the frequency of acquisition was set to 100 Hz.

Thirteen markers with a diameter of 10 mm were placed on the following landmarks on the patients’ bodies: six on the trunk (right and left acromion, clavicle, sternum, seventh cervical vertebra, and tenth thoracic vertebra), and seven on the affected upper limb (upper arm, lateral epicondyle, forearm, styloid processes of the ulna and the radius, and proximal heads of the second and the fifth metacarpal bones). Moreover, a marker was placed on the medial epicondyle in the static trial only (described below). In addition, three markers were placed on the glass.

Each patient was positioned with both hands on the table and the glass situated 400 mm from the table’s edge in the sagittal plane of the subject. First, a static trial was acquired. During the static acquisition, the subject remained still for about 2 to 5 s, while the markers’ positions in space were obtained. This acquisition enabled the calculation of the reference systems associated with the bone segments (local reference systems) and the markers’ coordinates in these reference systems.

Then, after a “go” signal, each participant was asked to reach for the glass with the affected limb, grasp it, bring it to the mouth, drink from it, and finally place it back on the table in the starting position. For each timepoint, i.e., before (T0) and after (T1) the 30-session rehabilitation treatment, the drinking task was repeated five times. A familiarization trial, not acquired, was allowed to explain the task and to allow patients to familiarize themselves with it.

### 2.5. Data Analysis

The markers’ trajectories measured using the stereophotogrammetric system were imported into MATLAB (MathWorks Inc., Natick, MA, USA); a low-pass filter was applied (6 Hz cut-off, 5th order, zero-lag, Butterworth filter), and spline interpolation was used to fill in the gaps when the markers were hidden for short periods.

**Figure 1:**
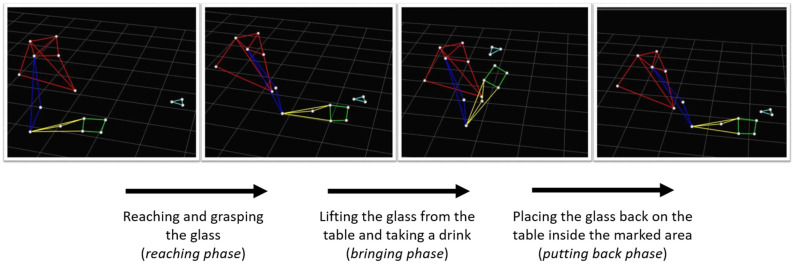
The investigated motor task with the three analyzed phases (reaching, bringing, and putting back).

Five phases were then identified: (1) reaching for the glass, (2) grasping it, (3) forward transport of the glass to the mouth, (4) drinking, and (5) back transport of the glass to the table. The onset and offset of each phase involving a limb displacement (1, 3, and 5) were determined according to the hand tangential velocity, i.e., as the instant when the tangential velocity of the hand markers first exceeded 2% of the maximum velocity in the analyzed phase, and the instant where the velocity remained below the same thresholds. Accordingly, the static phases (i.e., 2 and 4) were defined, as reported in Figure 1. Starting from the markers’ positions, the angular displacements of the trunk, shoulder, and elbow joints were then computed. Moreover, the positions of the four markers placed on the styloid processes of the ulna and the radius and heads of the metacarpals were averaged and used as hand positions for further analysis.

### 2.6. Quantitative Indices

We calculated quantitative indices associated with phases involving upper limb displacements, such as reaching for the glass, bringing the glass to the mouth, and putting the glass back on the table.

The investigated indices, computed for each phase separately, were selected according to the literature [18] and included:

- The duration of the task, to assess efficiency;
- The length ratio, to assess accuracy;
- The time to peak, to assess planning;
- The max speed;
- The log-dimensionless Jerk (LDLJ) and the Spectral Arc Length (SPARC), to assess smoothness [30]
- The range of motion of the shoulder and elbow and the trunk displacement, to assess spatial posture.

Except for the metrics related to spatial posture, all of the other metrics were computed using the position of the hand, as previously defined. Each reported kinematic metric was assigned to one construct based on its physiological interpretation [18]. The definitions of each index are reported in Table 1.

### 2.7. Statistical Analysis

Baseline data were compared between the two groups using the Mann–Whitney U test, or the Fisher’s exact test. The Mann–Whitney U test was also used to compare the absolute changes in the clinical scales, owing to the ordinal nature of the variables. A 2 × 2 mixed-ANOVA test, with *time* (two levels: T0 and T1) as within factor and *group* (two levels: robotic group and conventional group) as between factor was used to compare the improvement in the instrumental indices after rehabilitation between the two groups. For the statistical analysis, we used the mean values obtained from the five repetitions performed for each patient and each timepoint. A *p*-value lower than 0.05 was considered to be statistically significant. Statistical analysis was conducted using IBM SPSS Statistics software (version 28, IBM Corp., Armonk, NY, USA).

## 3. Results

### 3.1. Description of the Sample

Table 2 lists the demographic and clinical features of the sample population. The two groups were comparable in terms of their demographic and clinical characteristics.

### 3.2. Clinical Assessment

After the treatment, both groups improved in terms of upper limb motor function and strength, as well as in the activities of daily living and mobility, without differences between them, as reported in Table 3.

### 3.3. Instrumental Assessment

**Figure 2:**
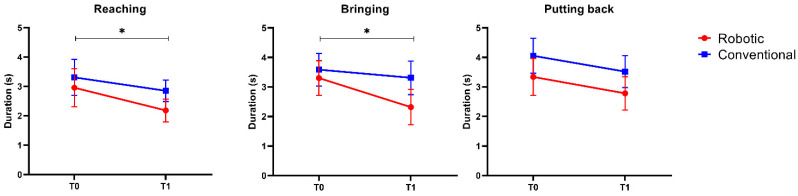
Values of the Duration index (efficiency) before (T0) and after (T1) the 30-session rehabilitation treatment, for the 3 analyzed phases of the drinking tasks, separately. Error bars represent the standard error. The symbol * indicates that the within-factor *time* was statistically significant (*p* < 0.05).

**Figure 3:**
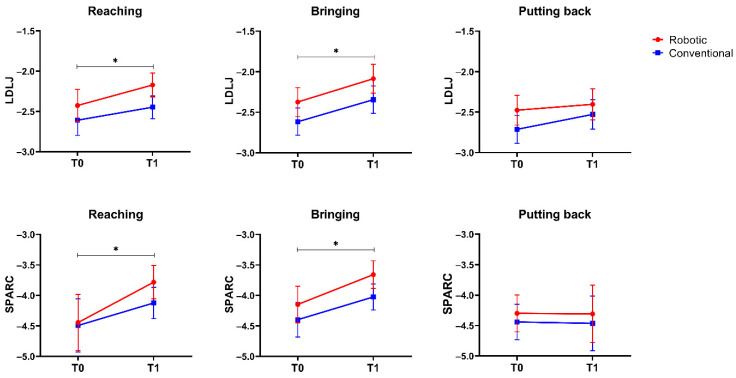
Values of the two metrics for smoothness evaluation, the log-dimensionless Jerk (LDLJ), and the Spectral Arc Length (SPARC), before (T0) and after (T1) the 30-session rehabilitation treatment, for the 3 analyzed phases of the drinking tasks, separately. Error bars represent the standard error. The symbol * indicates that the within-factor *time* was statistically significant (*p* < 0.05).

Table 4 reports the results of the instrumental evaluation. The statistical analysis showed that the interaction factor *time* × *group* was always not significant (*p* > 0.05), meaning that the evolution over time of the investigated indices did not differ between the two treatments. Concerning the main effect time, we found a statistically significant improvement in terms of efficiency (Figure 2) and smoothness (Figure 3) in both the reaching and the drinking phases. With respect to the effect size, the partial eta-squared values for the main effect time were as follows: 0.223 (duration, reaching phase), 0.198 (duration, bringing phase), 0.316 (LDLJ, reaching phase), 0.345 (LDLJ, bringing phase), 0.372 (SPARC, reaching phase), and 0.397 (SPARC, bringing phase), meaning a consistent large effect size. None of the investigated metrics showed a statistically significant change after the intervention in the putting back phase.

## 4. Discussion

In this study, we compared the improvement in a 3D upper limb movement in a sample of subacute stroke patients treated with either a robotic or a conventional approach. Specifically, we investigated a daily task, i.e., drinking from a glass, measured using an optoelectronic system.

Our results showed a similar improvement in the two investigated groups. These findings were consistent with larger RCTs that compared robotic and traditional upper limb treatment to restore dexterity after a stroke [14,15] and with the systematic review that found robotic-assisted arm training to be equivalent to conventional therapy [31]. Similarly, in the work of Chen et al. [32], the authors showed that RAT was non-inferior to therapist-mediated training in increasing arm capacity, activities of daily living, and social involvement, and superior in recovering from motor impairments.

These recent publications seemed to notice a possible higher effect of robotics on motor impairment recovery, but not in the activities of daily living. With this study, we aimed to understand if some differences in this domain could arise from a kinematic investigation of daily activity, but the changes in the analyzed parameters did not differ between the two groups. Nonetheless, our outcomes should not be interpreted negatively. In fact, in our sample, RAT was able to improve the patients’ ability to perform a daily living task similarly to the conventional treatment, even though the activities of daily living were not specifically addressed during the intervention. Additionally, robotic rehabilitation could provide benefits such as the ability to deliver standardized and repetitive training, real-time performance feedback, and the potential for increased patient motivation and engagement. Moreover, it is worthy to note that the use of robotics for rehabilitation is viewed as acceptable, useful, and beneficial by patients and healthcare professionals [33]. Finally, it was reported that by using an appropriate organizational model that optimizes the number of patients per therapist, as in our study, robotic therapy could have a better economic outcome than conventional therapy [34]. From this perspective, our results support the use of RAT in clinical practice.

Lencioni et al. [35] highlighted the effectiveness of RAT in the recovery of a 3D task using a quantitative technique on 32 post-stroke subjects treated with either a planar robot or a conventional approach. Specifically, the authors investigated the improvement in two 3D tasks (object placing and forearm pronation tasks) executed by means of a device for virtual reality and measured using an optoelectronic system. In this study, the post-stroke patients who underwent robotic rehabilitation showed greater increases in axial-to-proximal muscle synergies, which were linked to a significant improvement in proximal kinematics when compared to those who received the standard treatment. However, our results cannot be directly compared with those obtained in this study because of the different scenarios in which the tasks were performed (a real scenario in our case, a virtual scenario in the study of Lencioni et al.). In fact, it is worth noting that in a review [19], trials executed with virtual, robotic, or haptic devices were excluded because of the difficulty in comparing data and/or restriction of upper limb movements. Moreover, our task included a grasping phase that, even if not directly investigated, could have made the entire task harder to perform and hidden possible differences in the recovery of the two groups. In fact, a difference in recovery between reaching and grasping was observed in Lang et al. [36] and confirmed in the review of Saes et al. [37].

In our sample, after the treatments, the patients improved in terms of the efficiency of the gesture, as shown by a reduction in the time required to perform the first two phases, and similarly, an increase in the smoothness of the movement in the same phases. On the contrary, we did not observe a statistically significant improvement in terms of speed, accuracy, planning, or spatial posture. With respect to the reduction in the time required to perform the task and the increase in movement smoothness, these results were in accordance with the current literature. In fact, they were demonstrated to be the most responsive kinematic measures in patients with stroke [19], and well correlated with the clinical evaluation of motor impairment [38]. The ability to accomplish the task in a shorter period represents a gain in patient independence in everyday life, which is an essential issue in rehabilitation [39]. With respect to smoothness, it is a well-established biomarker of motor impairment in stroke patients. Several studies on upper limb disability after stroke have shown a loss in movement smoothness, i.e., an increase in movement intermittency [40,41], as well as an improvement after recovery [42]. After a stroke, upper limb movement seems to consist of a series of separate submovements [43], which may explain the observed lack of smoothness when compared to healthy participants executing the same motions. Furthermore, as a result of the rehabilitation intervention, these submovements gradually overlap and integrate to generate a more fluid movement over time [44]. When comparing multiple smoothness metrics, most of which are based on the jerk (the third derivative of the displacement) or the spectral arc length, the results are often inconsistent and, sometimes, opposite [45,46,47,48]. Regardless of the provided intervention, both the LDLJ and SPARC concurred in our research that patients showed a statistically significant improvement in movement smoothness after the rehabilitation in two out of the three analyzed phases.

The time to peak velocity and the length ratio, two additional measures depending on the hand kinematics, did not change after the interventions. It is worth noting that they were shown to be less sensitive to therapy in stroke patients than task duration or movement smoothness. In fact, similar to our result, the metric time to peak velocity in a previous study did not change over time [39], nor was longitudinally associated with FM-UE [39,49]. In the research conducted by Thrane et al. [23], the highest performance for time to peak velocity was attained 3 months after stroke, i.e., during an earlier phase post-stroke than our group, which was recruited about 110 days after the acute event. With respect to the length ratio, instead, it is worth noting that in the reaching phase, patients in the robotic group showed an increase of about 11% of the baseline value, while the control group did not show a similar trend (−1% of the baseline value). Accordingly, the *p*-value of the interaction factor *time* × *group* was approaching significance (*p* = 0.08). In fact, during RAT, the patients were required to perform and relearn straight reaching movements, with the help of a force field that constrained the trajectory inside a predefined path. Even though the statistical analysis did not demonstrate the advantage of this rehabilitation technique over the conventional intervention for this ability, our results suggested examining this motor feature in a larger sample to determine whether or not this trend will be confirmed.

With respect to the spatial posture, none of the investigated kinematic outcome measures changed after the treatments. Spatial abnormalities are also characteristics of motor impairment in patients after a stroke. In particular, it was suggested to include trunk movement in the 3D kinematic analysis of the tasks involving the upper limb, because trunk movement might combine with other kinematic factors and hide shoulder, elbow, or hand deficits [23]. In fact, even chronic patients rely excessively on the trunk to complete reaching activities [20,24]. Aprile et al. [24] demonstrated the different kinematic strategies employed by patients after a stroke while drinking. These strategies were either characterized by a significant increase in the forward displacement of the trunk and mouth in the reaching phase with a reduced arm elongation and no comparable backward displacement of the trunk and mouth in the bringing-to-mouth phase (the so-called strategy 1), or a significant increase in the forward displacement of the trunk and mouth in the reaching phase with a significant increase in the backward displacement during the phase in which the glass was brought closer to the mouth (the so-called strategy 2). Previous studies have shown that trunk displacement improved during the first three months after a stroke, but subsequent time points revealed only minor changes that were not statistically significant [21], confirming our results. Similarly, no statistically significant changes were found in arm abduction, even if a trend was detected (*p* < 0.07 in all three phases for the main effect *time*). Arm abduction was suggested to be incorporated, together with trunk movements, as crucial kinematics in stroke recovery and intervention experiments including reach activities, being a common synergetic muscle activity compensatory pattern, found to be non-physiological even 12 months after the acute event [23]. Taken together, these data seemed to suggest that post-stroke patients in the later phases of recovery (subacute and chronic phases) focused mostly on optimizing the acquired techniques (by enhancing the efficacy and smoothness of movement, as we saw) rather than altering them, even if both robotic and conventional intervention targeted the correctness and efficiency of the movements.

An unexpected result was the lack of significant improvement in the last analyzed phase, i.e., the phase in which patients had to place the glass on the table again after drinking. A possible explanation could be the lower significance of the phase with respect to the goal, i.e., drinking. It was probable that the patients were more attentive to the first two stages, which enabled them to achieve two distinct objectives (reach the glass and bring it to the mouth to drink). In contrast, the last phase was less specific (replace the glass on the table) and patients were likely less involved and focused on the task. The effects of goal-directed action (smoother, faster, more forceful, and more preplanned movement) were demonstrated in Trombly and Wu [50] and could help in the interpretation of our data on the last phase. However, to confirm this hypothesis, more specific studies on this aspect should be performed.

The main limitations of the study were the small sample size and that our analysis was based on a subset of patients who participated in a larger, randomized controlled trial that attempted to compare RAT and conventional therapy using clinical scales as primary outcomes. A further limitation was that our findings cannot be generalized to individuals who had a severe upper limb disability. Finally, other limitations included the lack of EMG analysis to better elucidate the neural mechanisms involved in the upper limb movement recovery, and the lack of a follow-up to better elucidate the retention of the detected improvements.

Nevertheless, these preliminary data confirmed that robotic rehabilitation was as effective as conventional rehabilitation in improving the ability to perform a functional task in terms of efficiency and smoothness. In light of previous research indicating that robotic therapy with an appropriate organizational model could have a better economic outcome than conventional therapy, and the equivalence between the two approaches in terms of motor outcome, we believe that our findings further support the implementation of robotics in clinical practice.

## Figures and Tables

**Table 1:** Quantitative indices used to quantify motor performance in the drinking task. Each index is computed for each phase separately.

Index	Construct	Definition
Duration	Efficiency	Time required to perform the phase of the task (calculated between the onset and the offset) and expressed in seconds
Length ratio	Accuracy	The ratio of the straight-line distance between the starting and final position of the hand and the distance traveled by the hand between the movement onset and offset
Time to peak	Planning	The time elapsing between the movement onset and the maximum speed, expressed in seconds.
Max speed	Speed	Peak speed of the hand during the movement, expressed in mm/s
Log-dimensionless jerk (LDLJ)	Smoothness	$LDLJ=-ln\left|\frac{{\left(t_{2}-t_{1}\right)}^{3}}{v_{peak}^{2}}\int_{t1}^{t2}{\left|\frac{d^{2}v\left(t\right)}{dt^{2}}\right|}^{2}dt\right|$ where *v(t)* is the speed of the hand, *t* is the time, t_1_ and t_2_ are the onset and the offset of the movement, and *v_peak_* is the peak speed of the hand
Spectral arc length (SPARC)	Smoothness	$SPARC=-\int_{0}^{\omega_{c}}{\left[{\left(\frac{1}{\omega_{c}}\right)}^{2}+{\left(\frac{d\hat{V}\left(\omega \right)}{d\omega }\right)}^{2}\right]}^{\frac{1}{2}}d\omega ;\hat{V}\left(\omega \right)=\frac{V\left(\omega \right)}{V\left(0\right)},$ where V(ω) is the Fourier amplitude spectrum of the speed v(t) of the hand, and $\hat{V}\left(\omega \right)\;$ is the normalized amplitude spectrum
Shoulder ROM	Spatial posture	Range of motion of the shoulder joint in the sagittal plane (flexion/extension movement)
Elbow ROM	Spatial posture	Range of motion of the elbow joint
Trunk displacement	Spatial posture	Range of motion of the marker placed on the sternum during the phase in the sagittal plane, expressed in mm

**Table 2:** Demographic and clinical features of the sample. Data are reported as mean (SD), or *n* (%), as appropriate. *p*-values refer to the Mann–Whitney U test or Fisher’s exact test, as appropriate.

Parameter	Robotic Group (*n* = 9)	Conventional Group (*n* = 10)	*p*-Value
Age (years)	64.9 (9.6)	69.0 (13.8)	0.315
Sex (male)	8 (88.9%)	7 (70.0%)	0.313
Time since stroke (days)	115.7 (58.4)	105. (31.7)	0.780
Fugl-Meyer Assessment for Upper Extremity	41.8 (7.9)	41.1 (7.5)	0.661
Motricity Index for Upper Extremity	61.1 (11.8)	59.7 (15.1)	>0.999
Modified Barthel Index	56.8 (22.4)	51.8 (19.3)	0.720

**Table 3:** Changes in clinical scales in the two treatment groups, together with the statistical analysis.

Parameter	Robotic Group (*n* = 9)	Conventional Group (*n* = 10)	*p*-Value
Changes in the Fugl-Meyer Assessment for Upper Extremity	9.4 (5.4)	10.4 (4.0)	0.549
Changes in the Motricity Index for Upper Extremity	11.1 (6.1)	13.0 (8.2)	0.604
Changes in the Modified Barthel Index	19.3 (20.1)	20.1 (15.5)	0.604

**Table 4:** Quantitative indices related to the investigated drinking task, for the two groups and the three phases, separately. *p*-values lower than 0.05 are highlighted in bold.

Quantitative Index with Timepoint		Reaching					Drinking					Putting Back				
		Robotic (Mean ± SD)	Conventional (Mean ± SD)	Time (*p*)	Group (*p*)	Time × Group (*p*)	Robotic (Mean ± SD)	Conventional (Mean ± SD)	Time (*p*)	Group (*p*)	Time × Group (*p*)	Robotic (Mean ± SD)	Conventional (Mean ± SD)	Time (*p*)	Group (*p*)	Time × Group (*p*)
Duration (s)	T0	2.96 ± 0.65	3.31 ± 0.61	**0.041**	0.464	0.580	3.3 ± 0.58	3.59 ± 0.55	**0.049**	0.409	0.265	3.34 ± 0.63	4.06 ± 0.59	0.127	0.346	0.976
	T1	2.19 ± 0.39	2.85 ± 0.37				2.32 ± 0.6	3.31 ± 0.57				2.78 ± 0.57	3.52 ± 0.54			
Max speed (mm/s^2^)	T0	393.65 ± 49.35	423.34 ± 46.82	0.987	0.497	0.769	434.86 ± 55.24	434.01 ± 52.41	0.109	0.876	0.760	527.04 ± 71.45	567.11 ± 67.78	0.701	0.767	0.720
	T1	385.74 ± 39.63	432.2 ± 37.59				484.19 ± 51.55	505.51 ± 48.91				562.13 ± 58.16	568.3 ± 55.17			
Length ratio	T0	0.76 ± 0.05	0.79 ± 0.05	0.171	0.746	0.080	0.89 ± 0.03	0.87 ± 0.03	0.262	0.400	0.429	0.85 ± 0.04	0.82 ± 0.04	0.380	0.768	0.478
	T1	0.85 ± 0.05	0.78 ± 0.05				0.94 ± 0.04	0.88 ± 0.04				0.85 ± 0.04	0.85 ± 0.04			
Time to peak speed (s)	T0	0.74 ± 0.21	0.98 ± 0.2	0.439	0.258	0.424	1.23 ± 0.19	0.98 ± 0.18	0.095	0.658	0.126	0.94 ± 0.15	0.81 ± 0.15	0.875	0.648	0.066
	T1	0.62 ± 0.17	0.98 ± 0.16				0.89 ± 0.14	0.96 ± 0.13				0.67 ± 0.25	1.04 ± 0.24			
LDLJ	T0	−2.43 ± 0.2	−2.61 ± 0.19	**0.011**	0.337	0.530	−2.37 ± 0.18	−2.62 ± 0.17	**0.005**	0.290	0.929	−2.48 ± 0.18	−2.71 ± 0.17	0.213	0.460	0.575
	T1	−2.17 ± 0.15	−2.45 ± 0.14				−2.09 ± 0.18	−2.35 ± 0.17				−2.4 ± 0.19	−2.53 ± 0.18			
SPARC	T0	−4.45 ± 0.46	−4.49 ± 0.44	**0.006**	0.699	0.381	−4.15 ± 0.3	−4.4 ± 0.29	**0.004**	0.381	0.660	−4.3 ± 0.3	−4.44 ± 0.29	0.954	0.760	0.981
	T1	−3.78 ± 0.27	−4.12 ± 0.26				−3.66 ± 0.23	−4.03 ± 0.21				−4.31 ± 0.47	−4.46 ± 0.45			
Shoulder flex ROM (°)	T0	38.1 ± 4.0	47.1 ± 3.8	0.972	0.042	0.741	13.8 ± 2.8	14.5 ± 2.6	0.224	0.897	0.907	12.19 ± 2.56	14.37 ± 2.43	0.208	0.437	0.986
	T1	39.1 ± 2.5	46.3 ± 2.4				17.1 ± 3.1	17.3 ± 2.9				14.7 ± 2.37	16.96 ± 2.24			
Shoulder ab/add ROM (°)	T0	12.8 ± 1.9	12.306 ± 2.0	0.064	0.714	0.067	12.0 ± 2.6	11.3 ± 2.7	0.055	0.839	0.931	12.3 ± 2.8	11.0 ± 2.9	0.067	0.714	0.893
	T1	9.9 ± 1.6	12.291 ± 1.7				9.4 ± 1.7	8.9 ± 1.7				9.7 ± 1.7	8.7 ± 1.8			
Elbow Flex ROM (°)	T0	31.7 ± 4.1	39.69 ± 3.9	0.903	0.309	0.512	58.8 ± 5.1	60.9 ± 4.9	0.390	0.693	0.835	62.8 ± 4.8	63.0 ± 4.5	0.465	0.907	0.852
	T1	34.3 ± 5.1	37.95 ± 4.9				60.7 ± 5.5	64 ± 5.2				64.6 ± 5.5	65.9 ± 5.2			
Trunk displacement (mm)	T0	136.0 ± 19.7	152.1 ± 18.7	0.281	0.396	0.814	117.4 ± 17.4	134.4 ± 16.6	0.122	0.228	0.413	134.5 ± 18.5	157.5 ± 17.6	0.380	0.358	0.834
	T1	146.9 ± 16.7	168.9 ± 15.9				129.9 ± 24.1	173.6 ± 22.8				152.9 ± 19.1	168.9 ± 18.8			

## Data Availability

Data are available from the corresponding author upon reasonable request.
